# Carboxyl-Terminal Truncations Alter the Activity of the Human α-Galactosidase A

**DOI:** 10.1371/journal.pone.0118341

**Published:** 2015-02-26

**Authors:** Mariam Meghdari, Nicholas Gao, Abass Abdullahi, Erin Stokes, David H. Calhoun

**Affiliations:** 1 Chemistry Dept., City College of New York, New York, NY, USA; 2 Biology & Medical Lab Technology, Bronx Community College, Bronx, NY, USA; Louisiana State University Health Sciences Center, UNITED STATES

## Abstract

Fabry disease is an X-linked inborn error of glycolipid metabolism caused by deficiency of the human lysosomal enzyme, α-galactosidase A (αGal), leading to strokes, myocardial infarctions, and terminal renal failure, often leading to death in the fourth or fifth decade of life. The enzyme is responsible for the hydrolysis of terminal α-galactoside linkages in various glycolipids. Enzyme replacement therapy (ERT) has been approved for the treatment of Fabry disease, but adverse reactions, including immune reactions, make it desirable to generate improved methods for ERT. One approach to circumvent these adverse reactions is the development of derivatives of the enzyme with more activity per mg. It was previously reported that carboxyl-terminal deletions of 2 to 10 amino acids led to increased activity of about 2 to 6-fold. However, this data was qualitative or semi-quantitative and relied on comparison of the amounts of mRNA present in Northern blots with αGal enzyme activity using a transient expression system in COS-1 cells. Here we follow up on this report by constructing and purifying mutant enzymes with deletions of 2, 4, 6, 8, and 10 C-terminal amino acids (Δ2, Δ4, Δ6, Δ8, Δ10) for unambiguous quantitative enzyme assays. The results reported here show that the *k*_*cat*_/*K*_*m*_ approximately doubles with deletions of 2, 4, 6 and 10 amino acids (0.8 to 1.7-fold effect) while a deletion of 8 amino acids decreases the *k*_*cat*_/*K*_*m*_ (7.2-fold effect). These results indicate that the mutated enzymes with increased activity constructed here would be expected to have a greater therapeutic effect on a per mg basis, and could therefore reduce the likelihood of adverse infusion related reactions in Fabry patients receiving ERT treatment. These results also illustrate the principle that *in vitro* mutagenesis can be used to generate αGal derivatives with improved enzyme activity.

## Introduction

Mutations in the *α*Gal gene result in the sphingolipidosis named Fabry disease [1]. The enzymatic defect is inherited as an X-linked disorder and is associated with a progressive deposition of the glycosphingolipids, including globotriaosylceramide, galabioasylceramide, and blood group B substance. In affected males this leads to early death due to occlusive disease of the heart, kidney, and brain.

De Duve [2] first suggested that ERT might be a successful approach to the treatment of lysosomal storage defects such as Gaucher’s and Fabry disease. For Gaucher’s disease, ERT produced unequivocal clinical responses [3, 4] that were subsequently confirmed by others [5–7]. Classical Fabry disease patients lack detectable levels of *α*Gal [1] so it should not be surprising that more than 80% of Fabry patients treated with agalsidase-beta [8] and more than 50% treated with agalsidase-alfa [9] developed an immune response. The antibodies produced are primarily of the IgG class and a fraction of the antibodies appear to exhibit neutralizing properties. These antibodies have been associated with an increase in urinary globotriaosylceramide levels due to the uptake of immune-enzyme complexes by granulocytes in the bloodstream and macrophages in the tissues [10–12].

ERT for Fabry disease patients was initially undertaken for males with the classic form of the disease (no detectable *α*Gal activity) in a variety of clinical trials [8, 9, 13–16], but therapy is now also underway for heterozygous females with Fabry disease [17–19] and is under consideration for children [20–22] and adults with atypical (low levels of enzyme) Fabry disease [23]. The two products used for ERT in Fabry disease patients have been compared [24]. The pattern of glycosylation on *α*Gal has been analyzed [25] and its importance for activity [26] and uptake by cells has been established [27, 28]. The limitations of current approaches for ERT for Fabry disease and the need for improved techniques have been discussed [10, 29, 30]. Efforts for gene therapy for Fabry disease are underway [31–38] and molecular chaperones are under investigation for specific alleles [39–41]. Substrate reduction therapy as an augmentation to ERT has been evaluated [42]. There are several reviews on the general topic of ERT for lysosomal storage diseases [43–47].

Expression of the human *α*Gal has been reported in *Escherichia coli* [48], baculovirus [49, 50] Chinese hamster ovary cells [51] and human foreskin fibroblasts [52]. The highest levels of heterologous *α*Gal expression was observed in *Pichia pastoris* [53]. Recombinant *α*Gal has also been produced in a modified strain of *Saccharomyces cerevisiae* that synthesized glycoprotein lacking the outer chain of N-glycan, a structure that is specific to yeast but not humans [28, 54]. When this *α*Gal was introduced into Fabry patient fibroblasts or a Fabry mouse model, there was hydrolysis of accumulated substrates [28, 54].

The methylotrophic yeast *P. pastoris* is the most highly developed of a small group of alternative yeast species chosen for their advantages over *S. cerevisiae* as expression hosts [55, 56]. Two attributes critical in its selection are the existence of well-established fermentation methods and the presence of the tightly regulated methanol-inducible promoter. AOX expression is undetectable by enzyme assay or mRNA production in cells cultured on carbon sources such as glycerol, but constitutes up to 30% of total soluble protein in methanol-grown cells. Heterologous genes under the control of the *P*_*AOX*1_ promoter can be maintained in an expression-off mode on a non-methanolic carbon source in order to minimize expression of potentially toxic heterologous proteins during cell growth. The *P. pastoris* expression system has now been successfully used to produce a number of heterologous proteins at commercially useful concentrations [57].

Lysosomal enzymes such as *α*Gal are glycoproteins that are modified in the Golgi to contain N- or O-linked carbohydrate structures [58]. The human *α*Gal is glycosylated at Asp residues 139, 193, and 215 [26] with branched carbohydrate structures that vary in composition and sequence depending upon the host species and tissue type [25]. For example, the enzyme purified from humans contains variable amounts (5–15%) of asparagine linked complex and high mannose oligosaccharide chains [1]. Consequently, multiple forms are present in SDS gels and in isoelectric focusing experiments that correspond to the plasma and various tissue forms. The recombinant human *α*Gal preparations used therapeutically are produced in human and CHO cells and these have distinct glycosylation patterns and differ in levels of sialic acid and mannose-6-phosphate [24]. The recombinant *α*Gal produced in insect cells [49, 50] and in *P. pastoris* [53] contain variable levels of mostly complex and high mannose side chains, respectively. Glycoproteins produced in *P. pastoris* typically contain from 6 to 14 mannose units (Man_6_GlcNac_2_ to Man_14_GlcNac_2_) that sometimes produces a Gaussian-like distribution of oligomannosides that may center near Man_12_GlcNac_2_ to Man_13_GlcNac_2_ [59].

These carbohydrate moieties serve a structural and functional role. For example, it has been demonstrated that glycosylation, particularly at Asn-215, is required for enzyme solubility [26]. Also, uptake of the enzyme by cells in vivo is affected by terminal mannose-6-phosphate residues on the enzyme [27], and the 10–12 sialic acid residues on the plasma form of the enzyme accounts for the prolonged circulatory half-life of the enzyme compared to the tissue form with only one or two sialic acid residues [60]. The identification of these multiple forms as derivatives of the same protein in purified enzyme preparations can conveniently be monitored by treatment with specific N-glycosidases or by Western blots.

Fabry disease patients with adverse reactions to the infusions are currently treated with antihistamines and antipyretics and the initial immune response has been manageable to date [61, 62], but it can be anticipated that life-long treatment required for these patients will lead to unacceptable levels of neutralizing antibodies. In this context it is reasonable to devise approaches to circumvent these adverse reactions and the development of derivatives of the enzyme with more activity per mg is a logical approach. Miyamura and coworkers [63] reported that carboxyl-terminal deletions of 2 to 10 amino acids of *α*Gal led to an increase in activity of about 4 to 6-fold as compared to wild type (WT). However, this data was qualitative or semi-quantitative and relied on comparison of the amounts of mRNA present in Northern blots to *α*Gal enzyme activity during transient infection of COS-1 cells. Here we use a *P. pastoris* expression system for the construction and purification of mutant enzymes with C-terminal deletions. The quantitative results reported here with purified enzymes reveal that C-terminal deletions results in an increase (Δ2, Δ4, Δ6, and Δ10) or decrease (Δ8) in enzyme activity.

## Materials and Methods

### Cell strains and plasmids

The *P. pastoris* host strain X-33 (No. K1740-01), *E. coli* strains TOP10 (No. C4040-50) and TOP10F′ (No. C665-11), plasmid pPICZ*α*A (No. K1740-01), and TOPO® XL PCR cloning kit (No. K4700-10) were purchased from Invitrogen.

### Bioreactor expression of recombinant **α**Gal in *P. pastoris*

High-cell-density fermentation was carried out as previously described [53] with a modified growth medium utilizing non-precipitating sodium hexametaphosphate as a phosphate source [64] and modified for a 7 L Applikon bioreactor. Fermentation medium of 3.5 L (0.93 g/l CaSO_4_, 18.2 g/l K_2_SO_4_, 14.9 g/l MgSO_4_.7 H_2_O, 9 g/l (NH_4)_2__SO_4_, 40.0 g/l glycerol) was autoclaved at 121°C for 20 min in the vessel. After cooling to room temperature, filter sterilized sodium hexametaphosphate (25 g/l of fermentation basal salt medium dissolved in 500 ml of deionized water) and 0.435% PTM1 trace elements (CuSO_4_.5 H_2_O 6.0 g, NaI 0.08 g, MgSO_4_.H_2_O 3.0 g, Na_2_MoO_4_.2 H_2_O 0.2 g, H_3_BO_3_ 0.02 g, CoCl_2_ 0.5 g, ZnCl_2_ 20.0 g, FeSO_4_.7 H_2_O 65.0 g, biotin 0.2 g, 5.0 ml H_2_SO_4_ per liter) were added to complete the fermentation medium. The pH was adjusted to 6.0 using ammonium hydroxide (28%).

Four frozen MGY cultures of 4 ml each were used to inoculate four 100 ml MGY cultures in 1-liter baffled flasks and grown at 250 rpm and 30°C until the OD600 reached 2 to 6. The cultivation was divided into three phases, the glycerol batch, glycerol-fed batch, and methanol-fed batch. The glycerol batch phase was initiated with 400 ml of inoculum shake-flask culture added to 4 L of the fermentation medium containing 4% glycerol and an initial value of 100% dissolved oxygen until a spike was observed indicating complete consumption of glycerol. Next, the glycerol-fed batch phase was initiated and a 50% w/v glycerol feed rate of 18.15 ml/h/liter initial fermentation volume and maintained until a cell yield of 180 to 220 g/liter wet cells was achieved. At this point the glycerol feed was terminated manually and a methanol-fed batch phase was initiated by starting a 100% methanol feed containing 12 ml PTM1 trace salts per liter. Methanol was initially fed at 3.6 ml/h/liter of initial fermentation volume, then increased to 7.3 ml/h/liter and finally increased to 10.9 ml/h/liter of initial fermentation volume for the remainder of the fermentation. Dissolved oxygen spikes were used during the glycerol-fed batch phase and methanol-fed batch phase and to monitor substrate levels. A dissolved oxygen level of 40%, pH of 6, and temperature of 25°C were maintained by an ADI 1030 regulator. Sampling was performed at the end of each phase and at lease twice daily and analyzed for cell wet weight and increased *α*Gal activity over time. Cultivation was terminated once a plateau in *α*Gal activity was observed.

### Construction of strains

**Fig 1:**
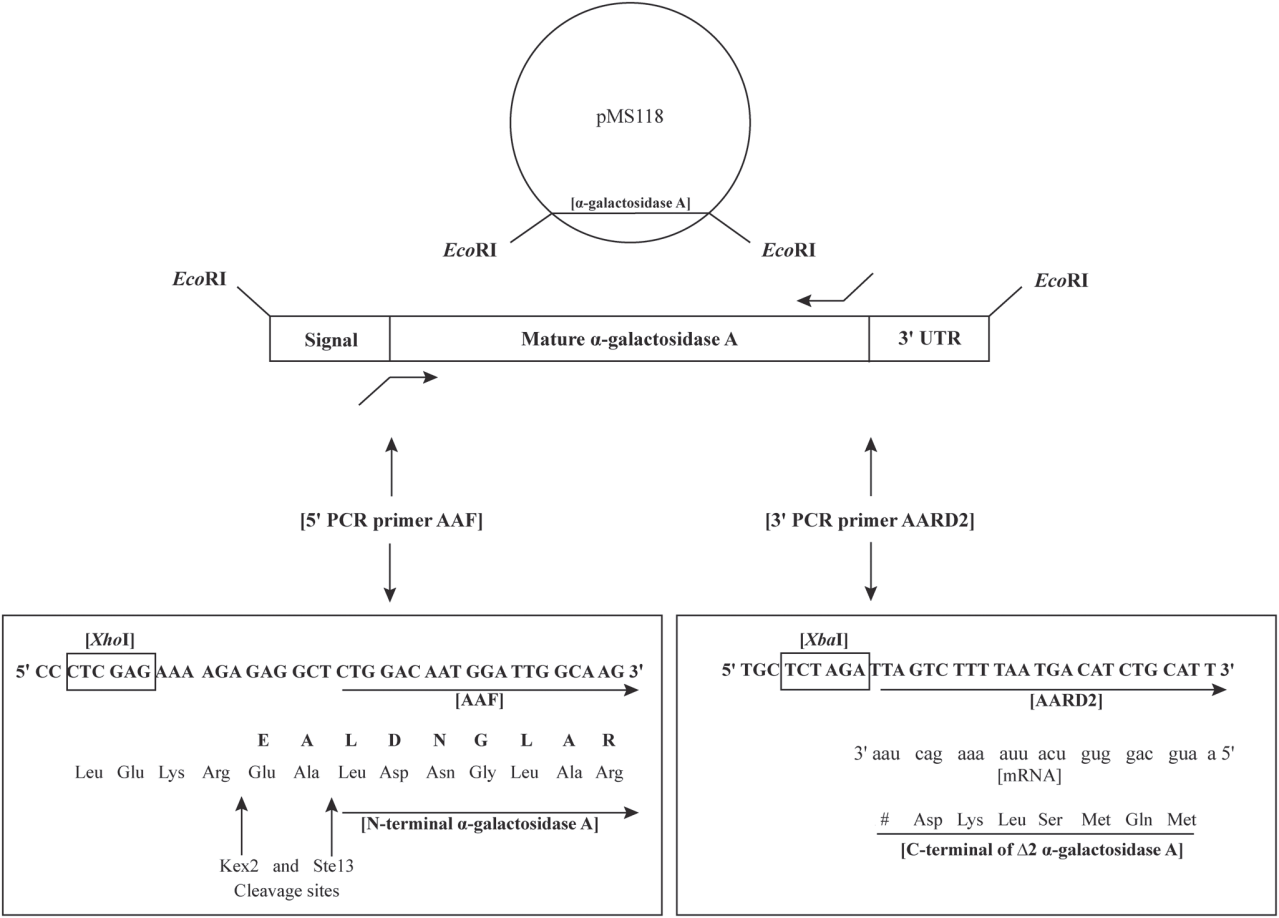
Introduction of a C-terminal deletion of 2 amino acids into **α**Gal. The strategy shown here for the Δ2 mutant was used to generate all five deletion mutations (Fig. 2). Plasmid pMS118 [48] contains the *α*Gal cDNA cloned as an *Eco*RI fragment to the *Eco*RI site of plasmid pUC9. Primers AAF and AARD2 (Fig. 2) were used as PCR primers for plasmid pMS118 DNA to generate cDNAs with a 5′ extension containing an *Xho*I site, Kex2 and Ste13 yeast signal cleavage sites, a 3′ end with an introduced *Xba*I site, and a deletion of C-terminal amino acids to generate the Δ2 mutant. Primer AAF anneals to the cDNA at the sequences encoding the N-terminal sequences of *α*Gal and primer AARD2 anneals to the C-terminal sequences of *α*Gal. Primer AARD2 anneals 12 nucleotides from the 3′ end of the cDNA and introduces a stop codon (UAA) after the aspartate codon three amino acids from the C-terminal end of the coding sequences of *α*Gal resulting in a deletion of the two C-terminal amino acids (Leu-Leu) of the human enzyme (right panel). Cloning to the *Xho*I and *Xba*I sites of plasmid pPICZ*α*A generates a protein fusion with the yeast signal peptide coding sequences in the vector. This signal peptide is removed by the Kex2 and Ste13 yeast signal peptidases through cleavage immediately upstream of the leucine corresponding the first amino acid of the mature form of *α*Gal (left panel). This strategy was generalized to create the other deletion mutants using the primers in Fig. 2. In the left panel, the N-terminal peptide LDNGLAR was identified in mass spectrometric analysis while EALDNGLAR was not (Fig. 5).

**Fig 2:**
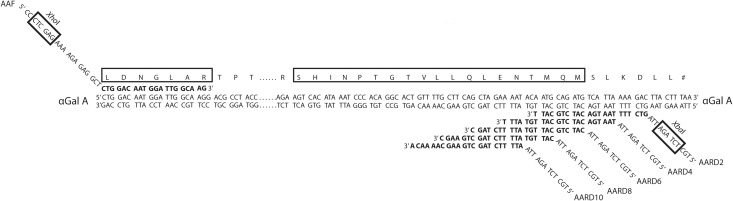
Primers and αGal cDNA used to generate Δ 2, Δ 4, Δ 6, Δ 8 and Δ 10 mutant cDNAs. DNA primers AAF, AARD2, AARD4, AARD6, AARD8 and AARD10 corresponding to the Δ2, Δ4, Δ6, Δ8 and Δ10 mutants were annealed to the cDNA of mature *α*Gal contained within pMS118 to generate 3′ end truncated PCR products for carboxy-terminal deleted enzymes. Primer AAF and primers AARD2 to AARD10 (indicated above) were annealed to the 5′ and 3′ ends of the cDNA, respectively. Primer AAF contains an *Xho*I site (indicated above) and partially encodes for a yeast signal peptide (see Fig. 1) to produce a fusion protein targeted for secretion from *P. pastoris*. Primers AARD2 to AARD10 were used to introduce an *Xba*I site (indicated above) and a premature UAA stop codon via an antisense ATT triplet immediately downstream of nucleotides complementary to *α*Gal (bold font) to produce cDNAs encoding for Δ2, Δ4, Δ6, Δ8, Δ10 mutants. The boxed LDNGLAR and SHINPTGTVLLQLENTMQM protein sequences (indicated above) are peptide fragments that were identified through mass spectrometry of the Δ6 mutant (Fig. 5).

Plasmid pMS118 [48] contains the *α*Gal cDNA cloned as an *Eco*RI fragment to the *Eco*RI site of plasmid pUC9. PCR primers (Fig. 1, 2) were used with plasmid pMS118 DNA and the PCR system (Roche, No. 11732641001) according to the vendor’s instructions. This generated cDNAs with a 5′ extension containing an *Xho*I site, Kex2 and Ste13 yeast signal cleavage sites, a 3′ end with an *Xba*I site, and a deletion of C-terminal amino acids to generate Δ2to Δ10 mutants (Fig. 1, 2). The PCR products were ligated to pCR-XL-TOPO to generate Δ2to Δ10 plasmids (Table 1). These plasmids were used for electroporation [53] into *E. coli* strain TOP10 or TOP10F′ (Table 1).

**Table 1:** Strains and Plasmids.

**Strain**	**Species**	**Plasmid**	**Description**
CC878	*E. coli*	pCC248	pCR-XL-TOPO derivative plasmid a modified cDNA using primers AAF and AARD4 to generate C-terminal deletion of 4 amino acids (Δ4)
CC892	*E. coli*	pCC262	pPICZ*α*A derivative plasmid with Δ4 cDNA insert
CC970	*E. coli*	pCC278	pCR-XL-TOPO derivative plasmid a modified cDNA using primers AAF and AARD6 to generate C-terminal deletion of 6 amino acids (Δ6)
CC973	*E. coli*	pCC281	pCR-XL-TOPO derivative plasmid a modified cDNA using primers AAF and AARD10 to generate C-terminal deletion of 10 amino acids (Δ10)
CC983	*E. coli*	pCC291	pPICZ*α*A derivative plasmid with Δ6 cDNA insert
CC990	*E. coli*	pCC298	pPICZ*α*A derivative plasmid with Δ10 cDNA insert
CC993	*E. coli*	pCC301	pCR-XL-TOPO derivative plasmid a modified cDNA using primers AAF and AARD2 to generate C-terminal deletion of 2 amino acids (Δ2)
CC994	*E. coli*	pCC302	pCR-XL-TOPO derivative plasmid a modified cDNA using primers AAF and AARD8 to generate C-terminal deletion of 8 amino acids (Δ8)
CC995	*E. coli*	pCC303	pPICZ*α*A derivative plasmid with Δ2 cDNA insert
CC997	*E. coli*	pCC305	pPICZ*α*A derivative plasmid with Δ8 cDNA insert
PC626	*P. pastoris*	pCC106	Integrated pPICZ*α*A derivative with WT cDNA insert [53]
PC897	*P. pastoris*	pCC262	Integrated pPICZ*α*A derivative with Δ4 cDNA insert
PC958	*P. pastoris*	pCC291	Integrated pPICZ*α*A derivative with Δ6 cDNA insert
PC960	*P. pastoris*	pCC298	Integrated pPICZ*α*A derivative with Δ10 cDNA insert
PC971	*P. pastoris*	pCC303	Integrated pPICZ*α*A derivative with Δ2 cDNA insert
PC973	*P. pastoris*	pCC305	Integrated pPICZ*α*A derivative with Δ8 cDNA insert
TOP10	*E. coli*	None	*E. coli* host for modified Δ2, Δ6, Δ8, Δ10 cDNA plasmids
TOP10F′	*E. coli*	None	*E. coli* host for pCC106 and modified Δ4 cDNA plasmid
X-33	*P. pastoris*	None	Expression host for *α*Gal expression

DNA sequence analysis using the universal M13 primers (Table 2) confirmed the expected insert for these pCR-XL-TOPO derivatives. The modified cDNAs were excised from pCR-XL-TOPO plasmids using *Xho*I and *Xba*I endonucleases and ligated into expression plasmid pPICZ*α*A treated with the same two restriction enzymes to generate expression plasmids (Table 1) that were subsequently used for electroporation [53] into strain TOP10 or TOP10F′ (Table 1). The nucleotide sequence of mutant cDNAs in pPICZ*α*A derivatives was analyzed (Genewiz) using 5′ AOX, 3′ AOX and *α*-factor sequencing primers (Table 2). These pPICZ*α*A derivatives were used for electroporation of *P. pastoris* strain X-33 to generate yeast expression strains (Table 1).

**Table 2:** Primers Used for DNA Sequence Analysis.

**Primer**	**Sequence**	**Function**
5′ AOX	5′ GACTGGTTCCAATTGACAAGC 3′	DNA sequencing primer for pPICZ*α*A
3′ AOX	5′ GCAAATGGCATTCTGACATCC 3′	DNA sequencing primer for pPICZ*α*A
*α*-factor	5′ TACTATTGCCAGCATTGCTGC 3′	DNA sequencing primer for pPICZ*α*A
M13: forward	5′ GTAAAACGACGGCCAG 3′	DNA sequencing primer for pCR-XL-TOPO
M13: reverse	5′ CAGGAAACAGCTATGAC 3′	DNA sequencing primer for pCR-XL-TOPO

Note. Primers were HPLC purified, 50 nmoles from Invitrogen

### Purification of **α**Gal using double affinity chromatography

Purification was as described [53, 65] with minor modifications (below). Bioreactor supernatant was passed through a 0.2 *μ*m hollow fiber filter (Spectrum Labs, No. M22M-300-01N) and subjected to diafiltration using a 50 kDa pore size hollow fiber filter (Spectrum Labs, No. M25S-300-01N) against wash buffer (0.1 M sodium acetate buffer, pH 6.0, 0.1 M NaCl, 1 mM MgCl_2_, 1 mM CaCl_2_, 1 mM MnCl_2_). The resulting supernatant was applied to a Con A Sepharose 4B (GE Healthcare No. 17-0440-01) column, pre-equilibrated with wash buffer, and washed with 5 column volumes of wash buffer. It was observed that near-saturating sugar eluent concentrations do not improve glycoprotein recovery as compared to lower concentrations and that elution phase pauses improve recovery [66]. In accordance with these findings, elution of *α*Gal was carried out using modified elution buffer I (0.5 M methyl-*α*-D-mannopyranoside, 0.25 M methyl-*α*-D-glucopyranoside in wash buffer) over 1.5 column volume blocks separated by 12-hour interval soaks. Elution was discontinued when the absorbance at 280 nm and enzyme assays showed negligible presence of protein and *α*Gal activity. No substantial difference in recovered enzyme was observed between purifications carried out with modified elution buffer I versus sugar saturated elution buffer I (data not shown). The Con A pool was subjected to diafiltration using a 50 kDa pore size hollow fiber filter (Spectrum Labs, No. M25S-300-01N) against binding buffer (25 mM citrate-phosphate buffer, pH 4.8 containing 0.1 M NaCl).

The Con A pool was applied to an immobilized-D-galactose gel column (Thio-Gal, Pierce No. 20372) pre-equilibrated with binding buffer. The column was washed with 5 column volumes of binding buffer and *α*Gal was eluted with elution buffer II (25 mM citrate-phosphate buffer, pH 5.5, 0.1 M NaCl, 0.1 M D-galactose) over 1.5 column volume blocks separated by 12 hour soaks. Fractions were assayed for enzyme activity and protein concentration and a peak tube with high specific activity was chosen as the sample to be used in a substrate saturation curve.

### Electrophoresis analysis

Samples (8 *μg*) were mixed with an equal volume of reducing sample buffer (Bio-Rad Laemmli sample buffer with 5% β-mercaptoethanol) and heated for 5 minutes at 95°C before loading on a Mini-Protean TGX Precast Gel 4–20% (w/v) (Bio-Rad No. 456-1094). Bands were visualized by Coomassie blue staining via the modified Fairbanks protocol [67].

### Western blot analysis

Western blot analysis was performed using an anti-*α*Gal polyclonal antibody produced in chicken (Pierce/ThermoSci #PA1-9528) and horseradish peroxidase-conjugated anti-Chicken IgY antibody (Sigma #A9046). After SDS-PAGE (2 *μg* of samples loaded), the gel was incubated with a nitrocellulose membrane (Whatman, No. 10402594) for 15 minutes at room temperature in Transfer Buffer (48 mM Tris, 39 mM glycine, 20% MeOH, pH 9.2) and the proteins were then transferred to the nitrocellulose membrane using a Bio-Rad Trans Blot SD Semi-Dry Transfer Cell. The membrane was blocked with 8% (w/v) non-fat dried milk in PBST [10 mM Na_2_HPO_4_, 1.8 mM KH_2_PO_4_, 137 mM NaCl, 2.7 mM KCl and 0.2% Tween 20 (pH 7.4)] at room temperature for 20 minutes. The membrane was then treated with primary antibody diluted in a milk/blot solution [1% (w/v) non-fat dried PBST] for 2 h at room temperature with mild shaking. After rinsing with PBST solution, the membrane was treated for 1 h at room temperature with secondary antibody diluted in the milk/blot solution. Protein bands were visualized on Kodak BioMax XAR film (VWR #lB1651454) with a Konica SRX-101A processor.

### Enzyme and protein assays

Activity of *α*Gal was assayed using the synthetic substrate, 4-methylumbelliferyl-*α*-D-galactopyranoside (MUG) as described [53] with modifications to a microtiter plate format (below). Enzyme activity is measured in units/ml where one unit is defined as the amount of enzyme required to convert 1 nmole of MUG to 4-methylumbelliferone in one hour at 37°C. An aliquot of 3 *μ*l was added to 27 *μ*l of enzyme assay buffer (5 mM MUG in 40 mM sodium acetate buffer, pH 4.5). This mixture was incubated at 37°C and 10 *μ*l aliquots were taken at two time points and added to 290 *μ*l of 0.1 M diethylamine in a microtiter plate to stop the reaction. Typically time points were chosen as 1–4 minutes and values that were proportional to time were considered valid. The fluorescence of each sample was measured at an excitation wavelength of 365 nm and an emission wavelength of 450 nm using a Tecan Infinite F200 microtiter plate reader. A standard curve of 10 *μ*l of 0 – 0.5 nmol 4-methylumbelliferone dissolved in MeOH in 290 *μ*l of 0.1 M diethylamine was used to quantitate MUG cleavage at specific time intervals. Analysis of the effects of MeOH indicated no effect on the 4-methylumbelliferone standard curve.

For samples containing higher protein concentrations, the BioRad DC Protein Assay (No. 500-0116) with a standard curve of (0.2 – 1.5) mg/ml was used according to the manufacturer’s specifications. For dilute samples of purified *α*Gal, a more sensitive fluorescence-based fluorescamine assay [68] with a standard curve containing lower protein concentrations of (4.0–160) *μ*g/ml was used. Briefly, 150 *μ*l of 0.05 M sodium phosphate buffer and 50 *μ*l of 1.08 mM fluorescamine dissolved in acetone were added to an aliquot of 50 *μ*l of the sample and standards, mixed and incubated for 12 minutes. The fluorescence of each sample was measured at an excitation wavelength of 400 nm and an emission wavelength of 460 nm. Bovine serum albumin (Bio-Rad No. 500-0112) was used as the standard in both assays. Absorbance and fluorescence measurements were conducted on a Tecan Infinite F200 microplate reader using 96-well plates.

### Mass spectrometry of a purified mutant enzyme

The Δ6 mutant was selected for mass spectrometry analysis conducted at the Rockefeller University Proteomics Resource Center in collaboration with M.T. Mark. SDS-PAGE gel slices were washed, de-stained, reduced using 10 mM dithiothreitol, alkylated using 100 mM iodoacetamide, and digested using trypsin. Peptides were then extracted from the gel two times, dried, and re-suspended in a 5% acetonitrile and 2% formic acid mixture. One third of each sample was loaded onto a C18 PepMap1000 micro-precolumn (300 *μ*m I.D., 5 mm length, 5 *μ*m beads, Thermo Scientific) at a flow-rate of 5 *μ*l/min, and subsequently onto an analytical C18 column (75 *μ*m I.D., 3 *μ*m beads, Nikkyo Technos Co.) at a flow rate of 300 nl/min. The gradient was 40 min long in the range 5 to 45% B (buffer A was 0.1% formic acid in water, and buffer B was 0.1% formic acid in acetonitrile). Eluted peptides were applied by electrospray directly into the LTQ-Orbitrap XL mass spectrometer from Thermo Scientific, operating in a 300 to 1800 m/z mass range. Tandem mass spectrometry was performed by collision induced dissociation using nitrogen as a collision gas. The resulting spectra were analyzed using Mascot and Proteome Discoverer 1.3 (Thermo Scientific) to identify the peptides in the sample.

### Thermostability and pH optimum of WT and mutant *α*Gal

Purified enzyme samples were diluted in 25 mM citrate-phosphate buffer, pH 5.5, 0.1 M NaCl, 0.01 M D-galactose. Samples of 50 *μ*l were incubated in triplicate at 50°C, 30°C and 40°C. Aliquots of 3 *μ*l were removed for enzyme assays every 15 minutes for two hours. Samples were assayed in 0.02 M citrate buffer, pH 3.0—pH 6.5, containing 2 mM MUG.

### Characterization of kinetic properties

Substrate saturation curves for *α*Gal have been reported using MUG at concentrations up to 2 mM, 5 mM, and 10 mM (in the presence of 0.1% BSA and 0.67% EtOH [24]). We noted that under our experimental conditions MUG is fully soluble at 2 mM, partially soluble at 5 mM, and chemically oversaturated at higher concentrations. Other investigators reported the use of sonication or detergent treatment to increase the solubility of MUG (e.g., [69]) but we avoided this approach in order to avoid potential artifacts due to the use of these techniques. Substrate saturation curves using 2 mM and 5 mM MUG as the highest concentrations were carried out and the kinetic parameters for *α*Gal were calculated separately obtaining similar values. The values reported here (Table 3a) were obtained using a substrate saturation curve of 0.3 to 2 mM MUG since this is the highest concentration that is fully soluble under our experimental conditions. The *K*_*m*_ and *V*_*max*_ values were calculated using Lineweaver-Burk and non-linear regression through the program Sigma-Plot (Systat Software, San Jose, CA).

**Table 3:** Values of *K*_*m*_, *V*_*max*_, *k*_*cat*_ and and the specificity constant (*k*_*cat*_/*K*_*m*_) for WT and C-Terminal Deletion Mutants of *α*Gal.

**A) MUG**				
**Comments**	**K_m_ (mM)**	**V_max_ (mmole/hr/mg)**	**k_cat_ (*s*^−1^)**	**k_cat_/K_m_ (**mM**^−1^ s** ^−1^)
WT	2.44 ± 0.44	3.36 ± 0.29	84.0	34.4
Δ2	4.52 ± 0.62	5.56 ± 0.73	139	30.8
Δ4	3.51 ± 0.29	7.29 ± 0.74	182	51.9
Δ6	4.21 ± 0.52	4.89 ± 0.32	122	29.0
Δ8	3.89 ± 0.27	0.742 ± 0.21	18.6	4.78
Δ10	2.96 ± 0.29	6.90 ± 0.71	173	58.3

**B) PNP*α*Gal**				
**Comments**	**K_m_ (mM)**	**V_max_ (mmole/hr/mg)**	**k_cat_ (*s*^−1^)**	**k_cat_/K_m_ (**mM**^−1^ s** ^−1^)
WT	15.0 ± 2.0	2.51 ± 0.17	62.8	4.18
Δ2	13.3 ± 1.2	5.14 ± 0.82	128	9.65
Δ4	15.7 ± 1.0	5.74 ± 0.39	143	9.13
Δ6	13.4 ± 1.1	1.89 ± 0.23	47.3	3.53
Δ8	13.0 ± 1.5	0.68 ± 0.08	17.0	1.31
Δ10	17.0 ± 3.0	6.24 ± 0.12	156	9.18

Note. The values given are for the human enzyme purified from *P. pastoris* and assayed in triplicate followed by Lineweaver-Burk and non-linear regression analysis. Comparison of both Lineweaver-Burk and non-linear regression kinetic parameters show good general agreement (data not shown). Non-linear regression results are displayed above. The *k*_*cat*_ was calculated using 90 kDa as the MW of *α*Gal. A) MUG was used as the substrate for enzyme assay. Mean and standard deviation measurements are from multiple assays of three independent enzyme preparations for the Δ8 enzyme, two independent enzyme preparations for the WT enzyme, and single enzyme preparations for the other mutant enzymes. B) PNP*α*Gal was used as the substrate for enzyme assay.

Kinetic parameters were also determined using the colorimetric substrate, para-nitrophenyl-*α*-D-galactopyranoside (PNP*α*Gal) [70]. Purified enzymes were diluted to approximately 20,000 units/mL as determined by fluorescent MUG assay. These diluted samples were then added at a proportion of 1:9 citrate-phosphate buffer (0.1 M) containing 7 – 50 mM PNP*α*Gal. Aliquots of 20 *μ*l of the enzymatic reaction were removed at 15 minute intervals to terminate the reaction over the course of an hour and added to 320 *μ*l of borate buffer (pH 9.8) in a microplate [71]. Product formation was monitored by absorbance at 400 nm. Linear reaction velocities were observed for all measurements. A standard curve of 0–150 *μ*M p-nitrophenylate in borate buffer (pH 9.8) [71] was used to quantitate product formation. *K*_*m*_ and *V*_*max*_ parameters were determined through non-linear regression using Sigma-Plot (Systat Software, San Jose, CA).

### Protein structure analysis

The crystal structure of *α*Gal (PDB 1R47) was viewed and analyzed in PyMOL (Delano Scientific). The MSLDKLL and QMSLKDLL peptides corresponding to the last 7 or 8 C-terminal amino acids of *α*Gal were built in PyRosetta [72] and visualized in PyMOL [73]. Interatomic distances were measured using the PyMOL wizard distance command.

A homology model of the coffee bean *α*-galactosidase was generated on the Phyre2 server [74]. The primary sequence of coffee bean *α*-galactosidase (GenBank No. AAA33022.1) was set as the query. The crystal structure of rice *α*-galactosidase (73% sequence identity to coffee *α*-galactosidase, PDB# 1UAS) was set as the template. Superposition of the coffee homolog and human crystal structure of *α*Gal (PDB# 1R47) was conducted in PyMOL [73]. Primary sequence alignments were carried out in ClustalOmega (EMBL-EBI).

## Results

### Purification of WT and mutant **α**Gal

**Fig 3:**
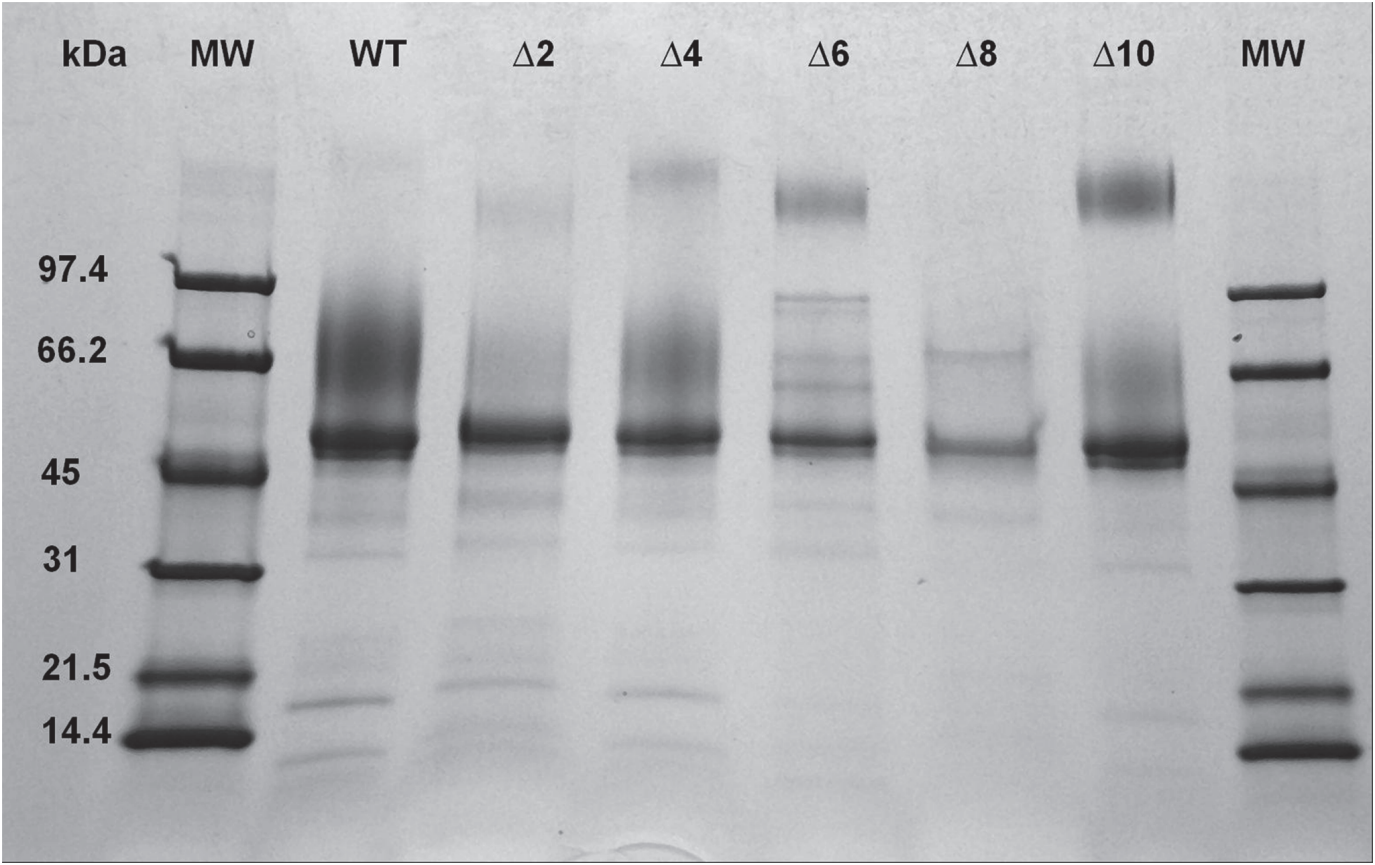
SDS-PAGE for purification of αGal. Purified samples were run on a 4–20% polyacrylamide gel, under reducing conditions, and stained with Coomassie Brilliant Blue. The contents of the lanes are as follows: molecular weight marker (lane 1 and 8), WT(PC626) (lane 2), Δ2 (PC995) (lane 3), Δ4 (PC897) (lane 4), Δ6 (PC958) (lane 5), Δ8 (PC973) (lane 6), Δ10 (PC960) (lane 7). The minor bands present in the purified fraction are consistent with high molecular weight glycoforms seen previously when WT enzyme was purified from the same *P. pastoris* expression system [53].

The WT and mutant enzymes were obtained from a 7 L bioreactor and purified (Table 4, Fig. 3) using Con A and Thio-Gal tandem affinity chromatography. This two column purification is simpler and faster than our previous purification methods that used three [50] or four [49, 53] columns and the yield, degree of purity, and final specific activities were similar for all three methods.

**Table 4:** Purification Table for WT *α*Gal Expressed in *P. pastoris*.

**Step**	**Total Protein (mg)**	**Total Activity Units × 10^6^)**	**Specific Activity Units/mg × 10^3^)**	**Purification (Fold)**	**Yield (%)**
Supernatant	10,928	134	610	1.0	100
Con A Pool	138	30.4	221	18.1	22.8
Thio-Gal Pool	4.18	15.7	3,771	309	11.8

Note. 5 mM MUG was used as the substrate for enzyme assay.

**Fig 4:**
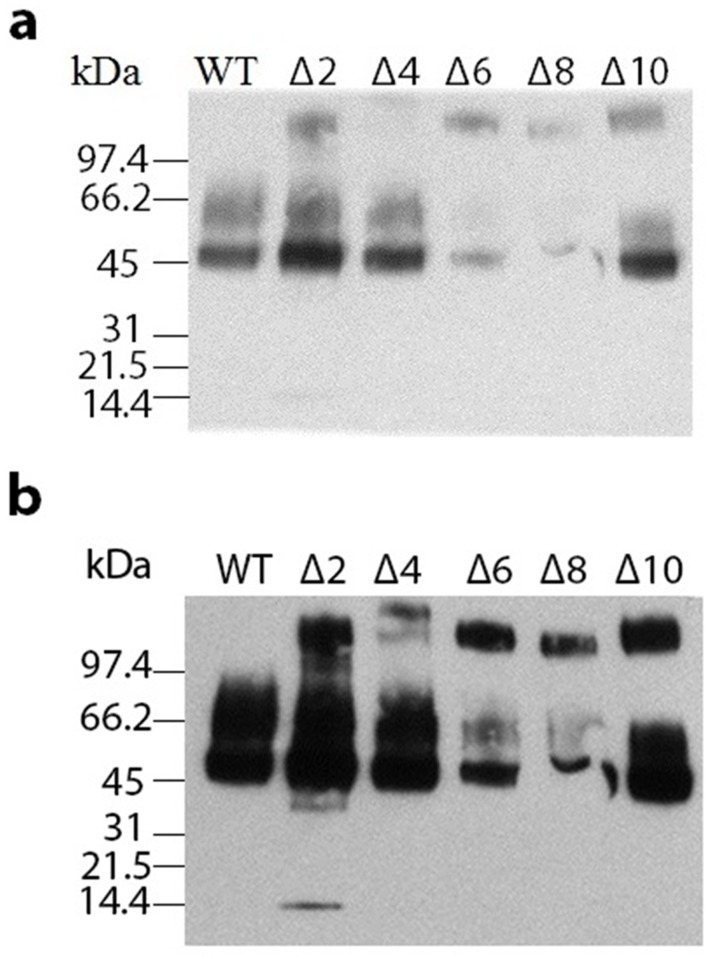
Western Blot of purified WT and mutant αGal. Purified WT and mutant enzymes were subjected to Western blotting using a polyclonal antibody raised against residues 55–64 and 396–407 of *α*Gal. (a) Blot at shorter and (b) longer exposure.

The non-glycosylated form of *α*Gal (41.8 kDa) is isolated from cells as multiple glycosylated species with a predominant band of about 50 kDa and multiple higher molecular weight forms that differ in extent of glycosylation (Fig. 3; See Introduction). We have previously demonstrated that high molecular weight glycoforms produced in insect cells and *P. pastoris* can be identified as derivatives of *α*Gal rather than contaminants and these glycoforms are converted to a single band on SDS gels of about 41.8 kDa with endoglycosidase treatment [49, 50, 53]. In this report we also use a Western blot (Fig. 4) to confirm that the high molecular weight forms seen on SDS gels for the WT and deletion mutants are all glycoforms of *α*Gal. In some cases lower molecular weight species present in purified enzyme preparations can be identified as *α*Gal fragments in Western blots (e.g., Fig. 4, lane 2). We quantitated the distribution of glycoforms in (Fig. 3, S1 Fig, online supplement) and there is no obvious correlation between the glycosylation pattern and catalytic activity. It is well established that glycosylation affects enzyme stability and enzyme uptake (above) but to our knowledge there is no evidence that the glycosylation pattern affects the catalytic properties of this enzyme.

### Mass spectrometry of a purified mutant enzyme

Two possible amino terminal amino acids, glutamate or leucine, could be produced in *P. pastoris* depending upon the selection of the signal peptidase, Kex2 or Ste13 (Fig. 1). Due to the fact that potential improper amino terminal processing may have an effect on kinetics, we selected one of the purified mutant enzymes (Δ6) for mass spectrometry analysis in order to identify the amino terminal sequence of this enzyme. This analysis also made it possible to provide independent verification of the expected changes in the C-terminal amino acid sequence predicted by *in vitro* mutagenesis (Fig. 1, 2).

**Fig 5:**
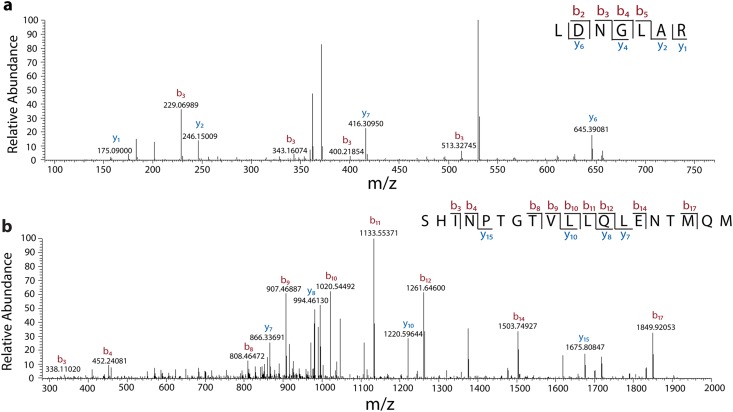
Mass spectrometry of purified Δ6 αGal. MS/MS spectra obtained from parental MS ions (a) m/z = 379.71 and (b) m/z = 1064.03 corresponding to a C-terminal and N-terminal peptide, respectively. Product ion peaks are annotated according to their predicted [M+H]^+^ forms. Annotations in red and blue correspond to b-series and y-series ion fragments, respectively.

The mature form of the enzyme (signal peptide removed; [75]) produced in humans begins with a leucine codon (Fig. 1, 2). Therefore, tandem mass spectrometry following tryptic digestion of the Δ6 *α*Gal purified from *P. pastoris* could produce tryptic peptides EALDNGLAR or LDNGLAR, depending upon the use of the Kex2 or Ste13 protease sites (Fig. 1, 2). A putative LDNGLAR peak was identified in the MS spectra with an m/z of 379.71, consistent with the (M+2H)^2+^ state of this peptide, while no peaks consistent with an EALDNGLAR peptide were found. We cannot eliminate what we consider to be the less likely possibility that the failure to detect the EALDNGLAR peptide may be due to the failure of the peptide to ionize in this MS experiment. Further fragmentation of the m/z = 379.71 associated peptide peak produced an MS/MS spectrum containing 4 of 7 possible y-ions and 4 of 7 possible b-ions from the expected fragmentation pattern of a hypothetical LDNGLAR peptide (Fig. 5a). This result indicates that the Ste13 signal peptidase of *P. pastoris* generates an enzyme with an amino terminus identical to the enzyme produced in humans.

A search for a Δ6 C-terminal tryptic peptide of SHINPTGTVLLQLENTMQM (Fig. 2) yielded a matching MS m/z = 1064.03 peak consistent with its (M+2H)^2+^ state. Further fragmentation also produced an MS/MS spectrum containing 4 of 19 possible y-ions and 9 of 19 possible b-ions consistent with the anticipated sequence (Fig. 5b). This result confirms the predicted C-terminal deletion of 6 amino acids and confirms the efficacy of the mutagenesis protocol used to produce this mutant enzymes.

Thus, the purified Δ6 *α*Gal mutant possesses an N-terminal sequence corresponding to the mature form of *α*Gal and a C-terminal sequence truncated by six amino acids.

### Thermostability and pH optima of WT and deletion mutants of **α**Gal

**Fig 6:**
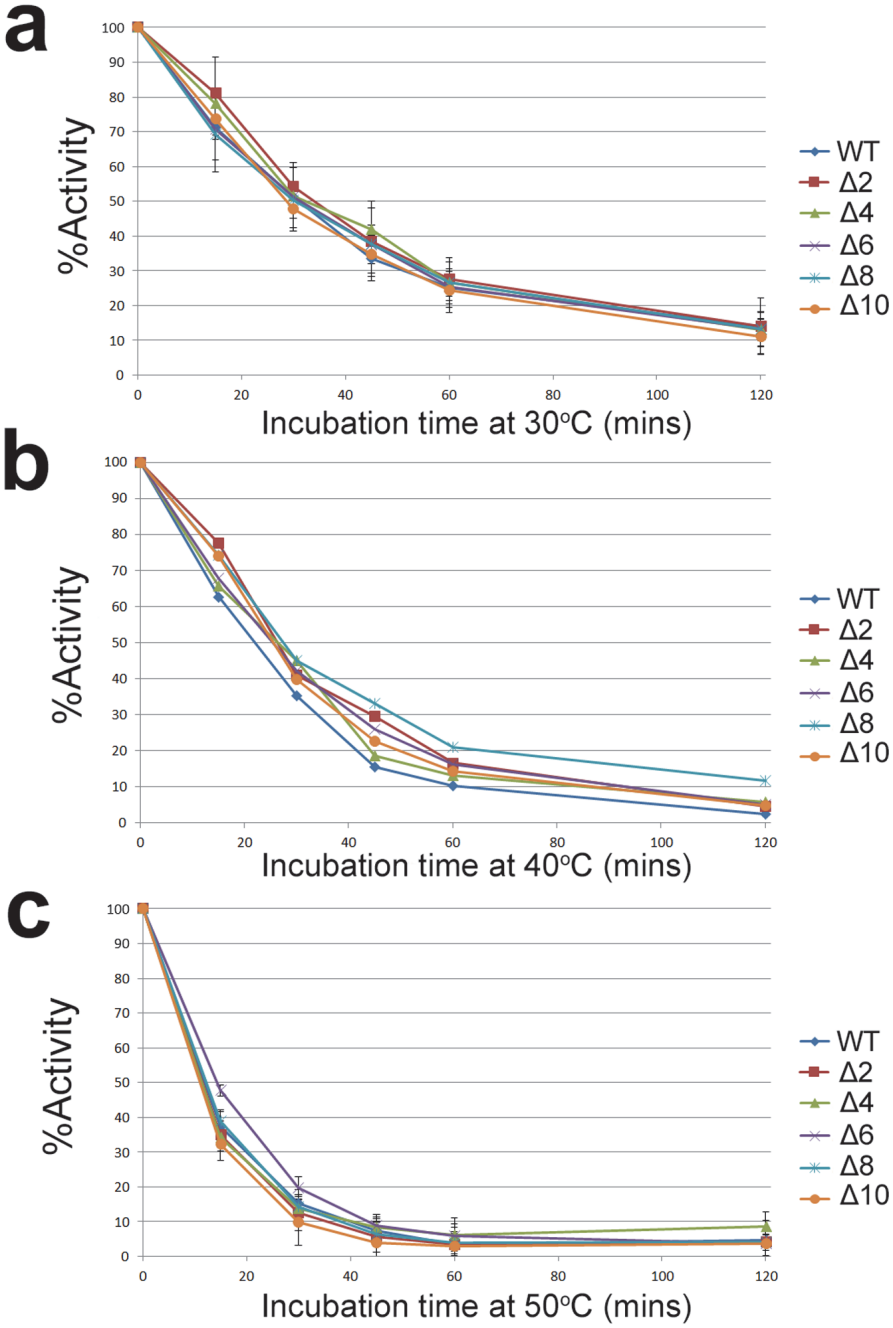
Thermostability profiles of WT and mutant αGal. Stability of recombinant WT and Δ2 to Δ10 mutant *α*Gal at 30°C (a), 40°C (b), and 50°C (c) at pH 5.5 as monitored by fluorescent enzyme assay. Initial activities ranged from approximately 300 to 1,900 units/mL for all enzymes assayed. % Activity is normalized against activity at t = 0 mins. Data points for (a) and (c) are the mean of a triplicate measurement with error bars equivalent to ± 1 standard deviation. Data points for (b) are the results of a single measurement. MUG was used as the substrate for enzyme assay.

**Fig 7:**
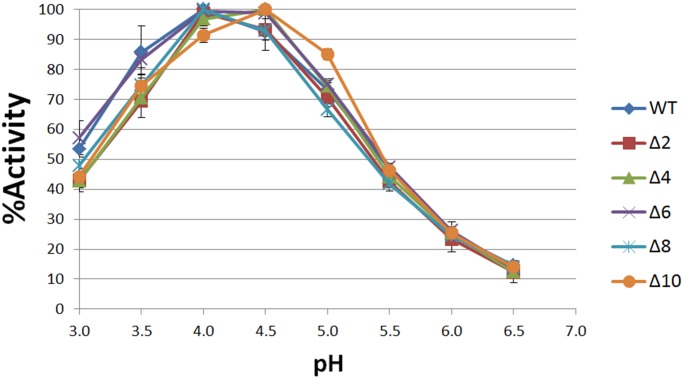
pH activity curves of WT and mutant αGal. pH activity curves for WT and Δ2 to Δ10 mutant *α*Gal. % Activity is normalized against each enzyme’s peak activity. Data points are the mean of a triplicate measurement and error bars are ± 1 standard deviation. MUG was used as the substrate for enzyme assay.

Preparations of purified WT and mutant *α*Gal show similar thermostability profiles at 30°C, 40°C, and 50°C, with activity half-lives of 30, 25 and 17 minutes, respectively (Fig. 6). The general trend of these profiles agree with previous results [76]. All enzymes show optimal activity near pH 4.5 (Fig. 7) in accord with previous reports for WT *α*Gal [70, 77–79], and there is no significant difference in the activity optima of purified WT and mutant *α*Gal.

### Kinetic analysis of WT and C-terminal deletion mutants

**Fig 8:**
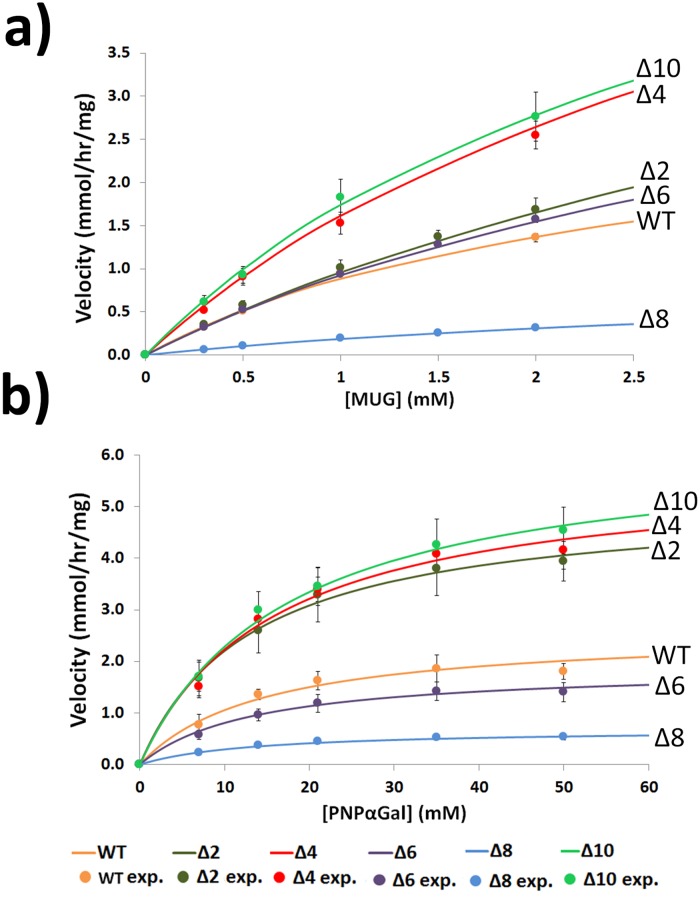
Substrate saturation curves of WT and mutant αGal. Purified WT, Δ2, Δ4, Δ6, Δ8and Δ10 *α*Gal were enzyme assayed in 0.3 to 2.0 mM MUG (Fig. 8a) and in 7 mM to 50 mM PNP*α*Gal (Fig. 8b) to measure initial velocities (mmol product per hr/mg enzyme). *K*_*m*_ and *V*_*max*_ parameters were extracted and compiled in Table 3. The figure indicates fits of Michaelis-Menten hyperbolas to experimental data indicated as mean ± one standard deviation.

The values for *K*_*m*_ and *V*_*max*_ for WT enzyme (Table 3a) are in accord with published values (Table 5). The range of *K*_*m*_ and *V*_*max*_ values for the enzymes purified from several sources in various laboratories over a period of more than 30 years (Table 5) are in good agreement and the observed subtle variations are in the range expected. However, more precision is expected for measurements recorded for enzymes purified from the same source in a single laboratory at one given time (Table 3a). Substrate saturation curves (Fig. 8a) and the calculated values for *K*_*m*_, *V*_*max*_, *k*_*cat*_, and *k*_*cat*_/*K*_*m*_ using the MUG substrate (Table 3a) reveal differences in the enzyme activity of the mutants compared to WT. Deletions of 2, 4, 6 and 10 amino acids approximately double the *k*_*cat*_/*K*_*m*_ (0.8 to 1.7-fold effect; 29/34.4 = 0.8 and 58.3/34.4 = 1.7) while a deletion of 8 amino acids decreases the *k*_*cat*_/*K*_*m*_ (7.2-fold effect; 34.4/4.78 = 7.2). There are corresponding changes in the *V*_*max*_ values and deletions of 2, 4, 6 and 10 amino acids approximately double the *V*_*max*_ (1.5 to 2.2-fold effect; 4.89/3.36 = 1.5 and 7.29/3.36 = 2.2) while a deletion of 8 amino acids decreases the *V*_*max*_ (4.5-fold effect; 3.36/0.742 = 4.5). There are also smaller differences in the *K*_*m*_ values of the C-terminal deletion mutants compared to the WT (Table 3a). The *V*_*max*_ data presented for the Δ8 (0.742 ± 0.21) and WT (3.36 ± 0.29) are derived from multiple assays from three and two independent enzyme samples, respectively, and this indicates the reliability of this data and adds strength to the interpretations of the data from the single enzyme preparations used for the other deletion mutants.

**Table 5:** Literature Values for *K*_*m*_ and *V*_*max*_ for the WT Human *α*Gal.

**K_m_ (mM)**	**V_max_ (mmole/hr/mg)**	**Source**	**Reference**	**year**
1.6	NA	Placenta	[80]	1978
2.9	1.7	Liver	[78]	1979
1.9	NA	Plasma	[60]	1979
2.5	NA	Spleen	[60]	1979
2.0	2.8	Spleen	[69]	1981
2.3	2.3	Sf9 insect cells	[81]	2000
2.0	4.8	Replagal	[24]	2003
2.0	4.8	Fabrazyme	[24]	2003
4.0	3.3	Fabrazyme	[82]	2009
2.8	2.6	COS-7 cells	[83]	2007
4.5	3.3	COS-7 cells	[84]	2011

Note. The values given are for the human enzyme purified directly from human tissues or from the indicated recombinant sources. Replagal is produced in human foreskin fibroblasts and Fabrazyme is produced in CHO cells. The average from these literature values are 2.6 ± 0.9mM (*K*_*m*_) and 3.2 ± 1.1mmole/hr/mg (*V*_*max*_). NA: not available. MUG was used as the substrate to determine the *K*_*m*_ and *V*_*max*_ values.

The effects of the C-terminal deletions on the kinetic properties of the enzyme using the artificial substrate MUG (Table 3a) could be due to alterations in the inherent catalytic mechanism of the enzyme [85]. Alternatively, the altered kinetic properties could be due to changes in the affinity of the enzyme for specific structural components of the artificial substrate, MUG. In this context, it is of interest to measure these kinetic parameters with an alternative substrate such as PNP*α*Gal. The results (Table 3b, Fig. 8b) indicate that there are similar changes in kinetic parameters using PNP*α*Gal as the substrate, including increases (2.2-fold effect; 9.18/4.18 = 2.2) and decreases (3.2-fold effect; 4.18/1.31 = 3.2) in the *k*_*cat*_/*K*_*m*_ for the specific C-terminal deletion mutants (Δ10 and Δ8, respectively). Taken together, these results suggest that the C-terminal deletions likely affect some aspect of the inherent catalytic mechanism of the enzyme.

## Discussion

This is the first report to establish in a quantitative manner that the C-terminal residues of *α*Gal act as a modulator of catalytic activity. Our results confirm the general results of Miyamura et al. [63] that C-terminal deletions of 2, 4, 6 and 10 amino acids increase the *k*_*cat*_/*K*_*m*_ compared to WT. However, our results differ in that we find that a deletion of 8 amino acids results in a decrease of *k*_*cat*_/*K*_*m*_. It should be noted that there are numerous experimental differences between these two reports. For example, our analysis used purified enzymes expressed in *P. pastoris* and their experiments measured *α*Gal enzyme activity during transient infection of COS-1 cells. There could be differences in *α*Gal mRNA or protein stability between *P. pastoris* and COS-1 cells, and other proteins in the cytoplasm of *P. pastoris* or COS-1 cells could interact directly or indirectly with the *α*Gal protein to affect its catalytic activity.

It is of interest that the two recombinant protein therapeutics, Fabrazyme (agalsidase-beta) and Replagal (agalsidase-alfa) contain C-terminal heterogeneity with truncated species lacking either one or two C-terminal residues [24]. Fabrazyme contains 69.7% full length protein with 7.6% Δ1 and 22.8% Δ2, while Replagal contains only 5.7% full length, with 73.2% Δ1 and 21.1% Δ2. These authors attribute the C-terminal heterogeneity to in vivo proteolytic processing of an undefined nature. These commercial enzymes do not differ significantly from the WT in *K*_*m*_ and *V*_*max*_ (Table 5) in spite of this degree of protein heterogeneity [24]. The significantly increased *V*_*max*_ of some of the mutants with C-terminal deletions suggests the basis for an improved treatment for Fabry disease.

These results also illustrate the principle that in vitro mutagenesis can be used to generate *α*Gal derivatives with improved enzyme activity. The potential for improved catalytic activity for this enzyme is illustrated by the existence of closely related enzymes with 3-fold to 250-fold higher activity (Table 6). A direct comparison of relevant amino acid residues between the human and related enzymes suggests the basis for rational in vitro mutagenesis to improve catalytic activity of the WT human enzyme. In this context it is likely that mutants which show altered catalysis against the MUG substrate used here also have a correspondingly higher rate of catalysis against the natural substrate, but this possibility needs to be tested directly.

Clinical trials for ERT show seroconversion frequencies of more than 50% for males treated with 0.2 mg/kg agalsidase-alfa [9] and 88% for 1.0 mg/kg agalsidase-beta [8, 86]. Increasing the dose of administered enzyme in seroconverted patients raised antibody titers in some patients [86, 87]. IgG antibody status shows a strong association with serious infusion associated adverse reactions [87]. IgG positive serum from Fabry patients exhibits *in vitro* neutralization of enzyme activity [88] and lessens targeting to key disease organs in a Fabry mouse model [89]. The disease marker of urinary globotriaosylceramide levels is increased in seropositive patients as compared to seronegative patients [86, 88]. It has been proposed that higher doses of administered enzyme could overcome the inhibitory effect of antibodies on treatment effectiveness [86]. By using an enzyme that is more active on a per mg basis, a therapeutic effect equivalent to WT is achievable through administration of a lower dose. Future studies should include examination of these mutant enzymes relative to WT in cultured cells [90] and in mouse models [91, 92].

The specificity constant (*k*_*cat*_/*K*_*m*_) has a maximum possible value determined by the frequency at which enzyme and substrate molecules collide in solution [93]. If every collision results in formation of an enzyme-substrate complex, diffusion theory predicts that *k*_*cat*_/*K*_*m*_ will attain a value of 10^8^ – 10^9^M^−1^s^−1^ [93]. The *k*_*cat*_/*K*_*m*_ of WT human *α*Gal is approximately 5.49 × 10^3^M^−1^s^−1^ (Table 6) suggesting the possibility that altered forms of the human enzyme may exist that have higher catalytic activity. A BLAST analysis [94] identified the 33 sequences most closely related to *α*Gal and kinetic parameters have been reported for 6 of these enzymes (Table 6). These enzymes share a high degree of sequence and structural similarities and are all in the same family 27 of glycosyl hydrolases [94]. There is a broad range in the values reported (Table 6) for *K*_*m*_, *k*_*cat*_ and *k*_*cat*_/*K*_*m*_. Thus, a detailed structural comparison of these enzymes may permit the identification of key amino acid residues that influence these kinetic parameters.

**Table 6:** Literature Values of *K*_*m*_, *k*_*cat*_, and the specificity constant (*k*_*cat*_/*K*_*m*_) for Glycosyl Hydrolase Family 27 *α*Gal Enzymes.

**Pubmed Accession code**	**Genus and species**	**Colloquial name**	**K_m_ (mM)**	**k_cat_ (s^−1^)**	**k_cat_/K_m_ (M^−1^ s^−1^)**	**Relative k_cat_/K_m_**	**Ref.**
NP_000160	*Homo sapiens*	Human	6.88	37.8	5.49 × 10^3^	1	[95]
NP_038491	*Mus musculus*	Mouse	1.40	N/A	N/A	N/A	[96]
WP_004844583.1	*Ruminococcus gnavus*	Bacteria	1.80	30.1	1.67 × 10^4^	3	[97]
AAC99325	*Saccharopolyspora erythraea*	Bacteria	0.650	23.3*	3.58 × 10^4^	6	[98]
P41947	*Saccharomyces cerevisiae*	Yeast	4.50	286	6.36 × 10^4^	12	[99]
BAB83765	*Clostridium josuil* (Catalytic Domain)	Bacteria	0.810	61.9*	7.64 × 10^4^	14	[100]
AAG24511	*Phanerochaete chrysosporium*	Fungus	0.198	272	1.37 × 10^6^	250	[101]

Note. PNP*α*Gal substrate was used to calculate kinetic values. Family 27 enzymes include the human *α*Gal and related enzymes in the CAZy database [102] that are most closely related as indicated by BLAST analysis [94]. *k_*cat*_ values for *S. erythraea* and *C. josuiI* were calculated based on the reported *V*_*max*_, and molecular weights.

**Fig 9:**
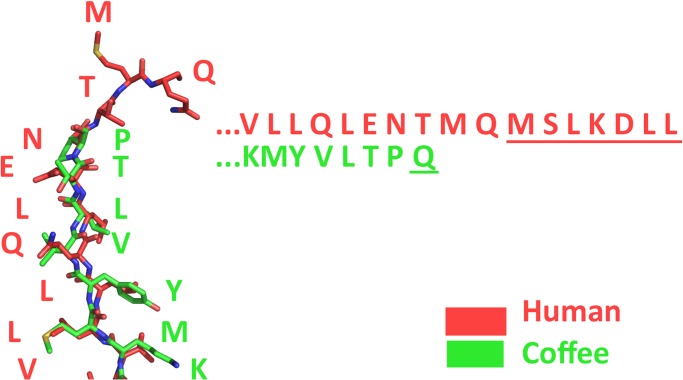
The C-termini of human and coffee *α*galactosidase. The crystal structure of human *α*Gal and a predicted model of the coffee homolog were superimposed. Underlined terminal residues, (MSLKDLL) in humans and (Q) in the coffee bean enzyme, indicate amino acids that could not be modeled due to conformational disorder. The terminal amino acid of the coffee enzyme (glutamine, Q) aligns with (threonine, T) in the human enzyme and is located 9 amino acids (MQMSLKDLL) from the C-terminus of the human enzyme.

Truncation of the C-terminus of the coffee bean *α*-galactosidase (Fig. 9) showed that deletion of one or two amino acids decreases activity and deleting 3 or more residues abolished activity completely [103]. The results with the coffee bean enzyme contrast those presented here for the human enzyme. Both results however demonstrate that the C-terminus of *α*Gal is critical for enzyme function.

We examined a superposition between the crystal structure of the human enzyme and a homology model of the coffee bean enzyme (see Methods) to explore a structural basis for this biochemical dissimilarity. The N-terminal catalytic (*α*/*β*)_8_ domain superimposes very well (RMSD of 0.729 *Å* over 1126 total atoms) while the C-terminal antiparallel *β*-domain superimposes poorly (RMSD of 2.494 *Å* over 342 total atoms). A primary sequence alignment shows a comparable trend; the catalytic domain shows high sequence conservation while the C-terminal domain does not. An alignment of the C-terminal ends of the human and coffee bean enzymes is presented (Fig. 9) indicating secondary structure alignment (left) and primary sequence alignment (right). If the C-terminal domain governs a conserved mechanism of enzymatic regulation across the human and coffee homologs, then the deleterious effect of removing C-terminal amino acids from the coffee enzyme is consistent with observations made by Miyamura et al. [63] on the human enzyme.

Because of the low sequence homology observed in the C-terminal residues it can be hypothesized that the effect on the catalytic activity due to C-terminus deletions in both the human and coffee bean *α*-galactosidase is due to disruption of the enzyme’s three-dimensional structure. This disruption could have an effect on enzyme dimerization, the ability to bind substrate, or potential interactions with other molecules in the cell.

The most straightforward expectation of a series of C-terminal deletions is a direct correlation between the extent of the deletion and the effect on enzyme activity. In this sense, the reduced activity of the Δ8 mutant relative to the other C-terminal deletion mutants (Table 3) is unanticipated. However, we note that similar effects have been reported by others who carried out C-terminal deletion studies, including the IN269 mutation for the integrase of HIV [104], the Δ8 and Δ9 mutants of the thymidine kinase of Epstein-Barr virus [105], and the D5 and D10 mutants of the plant vacullar H (+)-pyrophosphatase [106]. Differential proteolysis may also explain why the Δ8 construct does not follow the same trend as the other mutants.

**Fig 10:**
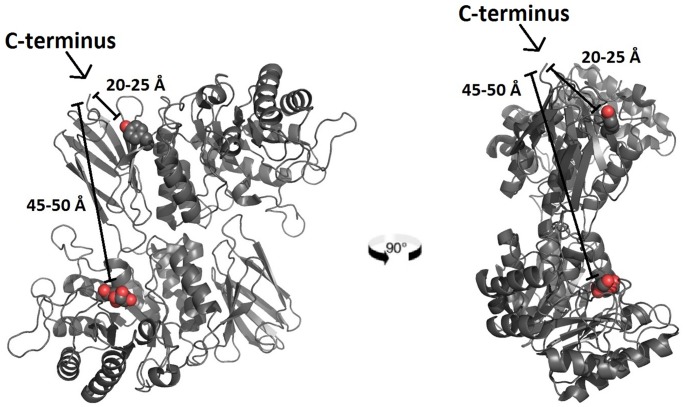
C-terminal Distance from Secondary Binding Site and Opposite Active Site. The homodimeric crystal structure of *α*Gal (PDB ID 1R47) solved by [94] is displayed in two different perspectives. Distance relationships relative to one out of the two possible C-termini are discussed. The carbon backbone is rendered in a ribbon format. The C-terminus on monomer A is separated by 20 *Å* to 25 *Å* from a secondary binding site for *β*-D-galactopyranose on the same monomer [85] which is marked by Tyr 329 rendered as spheres. The C-terminus on monomer A is also separated by 45 *Å* to 50 *Å* from the active site of monomer B which is marked by the *α*-D-galactopyranoside ligand also rendered as spheres.

The crystal structure of *α*Gal [94] revealed that the last visible residue of the C-terminus is separated by approximately 45 *Å* from the active site on the opposite monomer and is too far to have a direct effect on catalysis. However, within the same crystal structure (PDB 1R47) we measured C-terminal end to be only 20–25 *Å* away from a second ligand binding site for *β*-D-galactopyranose [85], demarcated by Tyr329 (Fig. 10). Directly visualizing a putative interaction between the C-terminus and the second binding site is not possible because C-terminal disorder limits resolution to the 7th or 8th amino acid from the full length C-terminal end. If these 7 or 8 amino acids were to adopt a fully-extended conformation, they would span a distance of 22–26 *Å* (see Methods), bringing the C-terminus within potential contact distance of the secondary binding site. A crucial missing detail is the functional significance of the secondary site. It may serve as a site for small molecule chaperoning [85]. It also might participate allosterically in a manner similar to phosphofructokinase-1, that is allosterically activated by ADP [107, 108], a product of the enzyme’s ATPase function. The ligand that binds the secondary site on *α*Gal is *β*-D-galactopyranose [85], which is the mutarotated product of the enzyme’s glycoside hydrolase function. A dynamic interplay may exist between the C-terminus, the secondary site and its ligand to affect the catalytic activity of *α*Gal through allostery or structural stability of the protein. Further mechanistic studies will be needed to work out the exact relationships between these putative components and their relevance to *in vitro* catalysis.

Due to the lack of direct contact between the carboxyl-terminal amino acids and the catalytic site of *α*Gal, the explanation for the effect of the deletions of the carboxyl-terminal amino acids is not obvious. Another hypothesis to be tested is that *α*Gal is in a class of enzymes like the *E. coli* dihydrofolate reductase [109–113] in which tunneling and coupled motion accounts for the effects of mutations distal from the catalytic site on enzyme function.

## Conclusions

C-terminal truncation mutants of *α*Gal were constructed, expressed and purified from *P. pastoris* using Con A and Thio-Gal affinity column chromatography. Michaelis-Menten parameters were measured on the purified mutants. Deletion of 2, 4, 6 and 10 amino acids approximately doubles *k*_*cat*_/*K*_*m*_ relative to WT (0.8-1.7-fold effect) while deleting 8 amino acids decreases *k*_*cat*_/*K*_*m*_ (7.2-fold effect). Mutants with increased activity are proposed as an improved alternative therapy over WT enzyme for Fabry disease patients. These results also illustrate the principle that *in vitro* mutagenesis can be used to generate *α*Gal derivatives with improved enzyme activity.

## Supporting Information

S1 Fig Quantification of Bands in SDS-PAGE. Band intensities of the SDS-PAGE in (Fig. 3) were quantified by Image Acquisition and Analysis software (VisionWorks®LS, UVP Inc., Upland, CA).
(TIF)
